# A “building block” approach to the new influenza A virus entry inhibitors with reduced cellular toxicities

**DOI:** 10.1038/srep22790

**Published:** 2016-03-08

**Authors:** Dongguo Lin, Fangfang Li, Qiuyi Wu, Xiangkun Xie, Wenjiao Wu, Jie Wu, Qing Chen, Shuwen Liu, Jian He

**Affiliations:** 1 School of Pharmaceutical Sciences, Southern Medical University, 1838 Guangzhou Avenue North; Guangzhou 510515, P. R. China; 2 Guangdong Provincial Center for Disease Control and Prevention, 160 Qunxian Road, Guangzhou 511430, P. R. China; 3 School of Public Health and Tropical Medicine, Southern Medical University, 1838 Guangzhou Avenue North; Guangzhou 510515, P. R. China

## Abstract

Influenza A virus (IAV) is a severe worldwide threat to public health and economic development that results in the emergence of drug-resistant or highly virulent strains. Therefore, it is imperative to develop potent anti-IAV drugs with different modes of action to currently available drugs. Herein, we show a new class of antiviral peptides generated by conjugating two known short antiviral peptides: part-1 (named Jp with the sequence of ARLPR) and part-2 (named Hp with the sequence of KKWK). The new peptides were thus created by hybridization of these two domains at C- and N- termini, respectively. The anti-IAV screening results identified that C20-Jp-Hp was the most potent peptide with IC_50_ value of 0.53 μM against A/Puerto Rico/8/34 (H1N1) strain. Interestingly, these new peptides display lower toxicities toward mammalian cells and higher therapeutic indices than their prototypes. In addition, the mechanism of action of C20-Jp-Hp was extensively investigated.

Influenza A viruses (IAVs) are one of the major causative pathogens of human acute respiratory disease responsible for seasonal epidemics and reoccurring pandemics of influenza, which poses a significant threat to human health and economic development. So far, there are only two classes of drugs available for the treatment of influenza A virus infection: the matrix protein 2 (M2) inhibitors such as amantadine and rimantadine, and the neuraminidase (NA) inhibitors like oseltamivir and zanamivir^1^. These clinically used drugs are functioned by blocking the proton channel activity of the influenza A viral M2 protein, or binding to NA to inhibit virus budding^2^. However, due to the emergence of drug-resistant viral strains, new antiviral strategies, targeting other viral proteins or cellular factors involved in the influenza virus life cycle, are urgently needed^3^.

With respect to the influenza A virus life cycle, the virus entry mediated by hemagglutinin (HA) is the first step for viral infection. HA is a viral surface glycoprotein consisting of two subunits: HA1 and HA2, linked by a single disulfide bond. In the events of virus entry, the HA1 subunit is responsible for binding the virus to sialic acid-containing receptors on host cells, while the HA2 subunit is for fusion which subsequently leading to viral endocytosis^4^,^5^,^6^. Given the critical role of HA in the process of viral infection, the HA including HA1 and HA2 subunits is a potential target for antiviral drug to intervene, thereby blocking the entry of virus into host cells^7^.

From phage-displayed random peptide libraries, Teruhiko Matsubara and his co-workers had identified an N-stearoyl lipopeptide of C18-ARLPR that was able to inhibit the replication of influenza A/Puerto Rico/8/34 (H1N1) and A/Aichi/2/68 (H3N2) with IC_50_ values of 1.9 and 1.6 μM, respectively^8^. The structure of this peptide was deduced to be the mimic of sialic acid, thus binding to the sialic acid-binding site in HA1 subunit of HA. As a result, this peptide might be used as a lead compound for novel antiviral drug discovery.

In our previous work^9^, by employing an H5N1 pseudo-virus based high-throughput screening approach, we discovered a peptide of C12-Hp as a lead for anti-IAV drug development. The experimental data and a docking simulation proposed that instead of interaction with sialic acid binding site of HA1, C12-Hp may interact with HA2 subunit to inhibit the fusion of virus with host cells. Further structure-activity relationship studies showed that the antiviral activity, as well as the selectivity index (SI) of this peptide was enhanced alongside the increase of the lengths of lipid chain, of which C20 fatty acid substituted congener (C20-Hp) exhibited the highest potency against tested viral strains. Nevertheless, the comparatively low SI value of 20 limited its application^9^.

To enhance the selectivity index of a drug candidate, the traditional approach is *via* an extensive structure-activity relationship study or a rational modification based on the 3D structural analyses of ligand-receptor interactions^10^,^11^. In this paper, we try to resort to a different avenue by using two functional peptides as “building blocks”, and then placing them at C- and N-termini respectively^12^. To make these domains more flexible, we respectively connect them with and without a -GGG- linker, thus generating a hybridized peptide library. With these extensive efforts, we expect that new antiviral peptides with modified biological properties would be created.

To fulfill this purpose, in this work, we employed a peptide of ARLPR (designated as Jp) as one domain^8^, while KKWK (designated as Hp) as the other domain^9^. A small combinatorial peptide library containing these two domains was thus generated (Table 1). As a consequence, antiviral activity screening against influenza strain of A/Puerto Rico/8/34 (H1N1) showed that new peptide of C20-Jp-Hp displayed the highest antiviral activity with the best selectivity. Furthermore, the mechanism study suggested that these peptides was represented as a new group of viral “entry blockers” by inhibiting the conformational changes of HA2 subunit, thereby blocking the entry of virus into host cells. In addition to providing novel antiviral agents as “entry inhibitors”, this paper proposes a promising approach to design new antiviral agents with high selective indices. Herein, we report on the design, antiviral activity and mode of action of these peptides.

## Results

### New virus “entry blockers” were constructed by conjugating two functional peptide domains

The new virus “entry inhibitor” was constructed by connecting two functional “blocks” of Jp and Hp at the C- and N- termini, respectively. Peptide Jp (sequence: ARLPR) was reported to mimic the structure of sialic acid and bind to sialic acid binding site in HA1 subunit^8^, while Hp (sequence: KKWK) was suggested to interact with HA2 subunit to inhibit the fusion of virus with host cell membrane^9^. To make these two domains more flexible, in addition to connecting them directly, we also conjugated them with a short peptide linker containing three glycine residues.

Our previous work has shown that the antiviral potency of KKWK was strongly associated with the lengths of lipid chain, which we concluded that the longer of the lipid chain was, the higher of the anti-IAV activity and selectivity index would be. Accordingly, peptide KKWK conjugated with C20 fatty acid displayed the best antiviral activity. On the basis of this progress, we thus selected C16, C18 and C20 fatty acids as the lipid tails, and respectively conjugated them to the N-termini of the newly generated peptides, as shown in Table 1.

**Figure 1:**
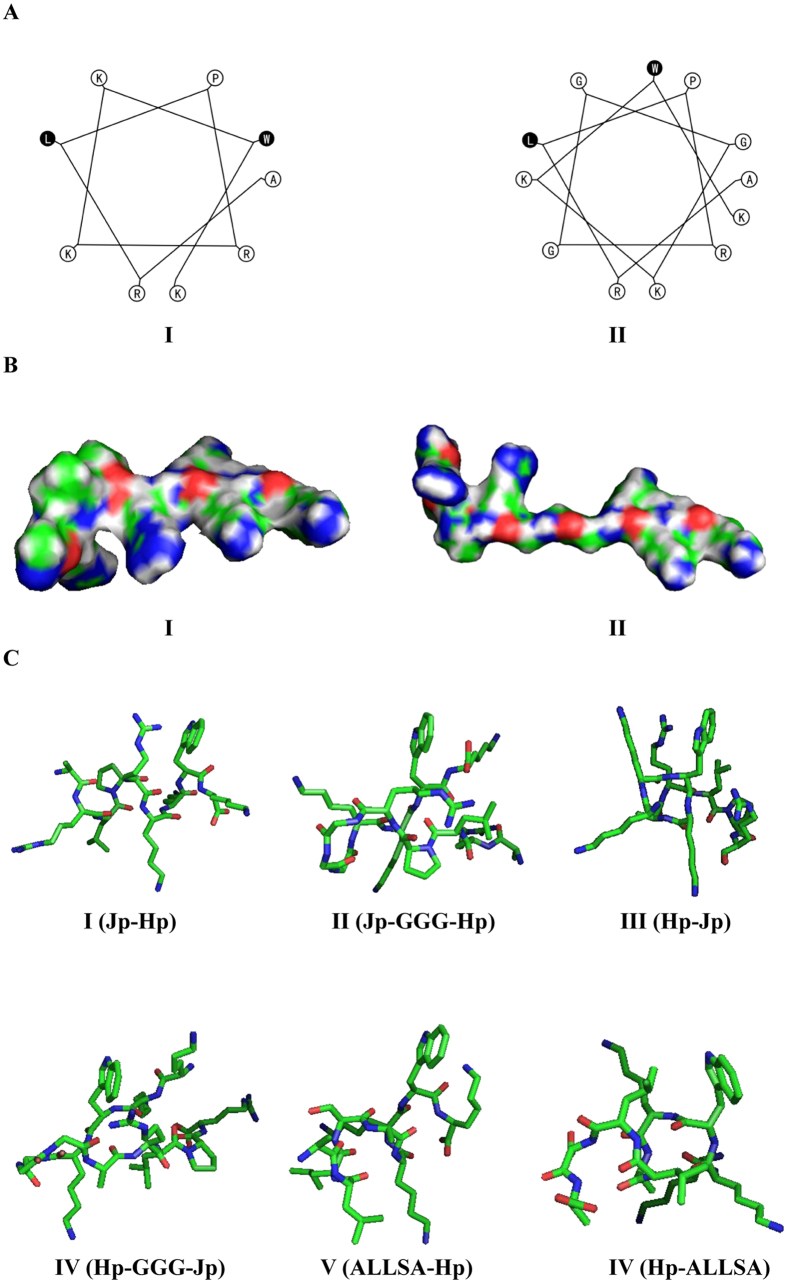
(**A)** The helical wheel projection of peptide Jp-Hp and Jp-GGG-Hp, with hydrophobic residues shaded. (**B)** Predicted 3D structures of Jp-Hp and Jp-GGG-Hp. The calculation of 3D structure was based on the web tool at: http://bioserv.rpbs.univ-paris-diderot.fr/PEP-FOLD/; (**C**) is the 3D structure of selected peptides, where the possible intramolecular interactions between the guanidinium group of argnine and the indole ring of tryptophan were observed in peptides C20-Jp-Hp (I) and C20-Hp-Jp (III), while these interactions were absent in other peptides. For all peptides in (**A–C)**, the number of I to VI refers to peptides Jp-Hp (I), Jp-GGG-Hp (II), Hp-Jp (III), Hp-GGG-Jp (IV), ALLSA-Hp (V) and Hp-ALLSA (VI), respectively.

The comparison of these new sequences indicates that C20-Jp-Hp appears to be more rigid and compact than C20-Jp-GGG-Hp, while the latter is more relaxed and flexible, which might be associated with the differences in their antiviral activities, as demonstrated in Fig. 1A,B.

### Peptide C20-Jp-Hp displays potent antiviral activities toward a broad influenza A viral strains

Next, we employed cytopathic effect (CPE) inhibition assay to evaluate the antiviral effects of these peptides against influenza strain of A/Puerto Rico/8/34 (H1N1). As shown in Table 1, peptides conjugated with C20 lipid chain exhibited the best activity toward tested viral strain, of which C20-Jp-Hp was the most potent one with the IC_50_ value of 0.53 μM, higher than its prototype of C18-Jp and C20-Hp. To our surprise, the peptides containing -GGG- linker showed a weaker activity than their congeners without the linker.

To confirm the antiviral effects of these peptides, by using the same screening method of CPE assay, several potent peptides were then selected to test a panel of other influenza viral strains including A/Aichi/2/68 (H3N2), A/FM/1/47 (H1N1) mouse adapted strain, and neuraminidase inhibitor-resistant strain of A/Puerto Rico/8/34 with NA-H274Y mutation^13^. In addition, three clinical isolates of 690 (H3 subtype), 699 (H3 subtype), and influenza B virus, currently circulating in Guangdong province, China, were also selected. The data in Table 2 showed that C20-Jp-Hp still was the most potent peptide against all tested strains including the drug-resistant strain of NA-H274Y mutation and clinically relevant viruses of 690, 699 and influenza B viruses (Table 2) with the IC_50_ values ranging from 0.5 to 2.0 μM, displaying a promising application in the development of anti-IAV therapy.

### The enhanced anti-IAV activity of C20-Jp-Hp is associated with the structure of Jp (ARLPR)

To weigh the contribution of Jp domain (ARLPR) to the antiviral effects of C20-Jp-Hp, we synthesized two peptides of ALLSA-Hp and Hp-ALLSA by replacing the Jp domain with a scramble peptide of ALLSA, and then tested their activities against influenza viruses, as indicated in Table 2. Consequently, the antiviral activities of C20-ALLSA-Hp and C20-Hp-ALLSA were close to C20-Jp-GGG-Hp and C20-Hp-GGG-Jp, while weaker than C20-Jp-Hp and C20-Hp-Jp (Table 2).

The different anti-IAV activities between C20-Jp-Hp and C20-ALLSA-Hp, as well as between C20-Jp-Hp and C20-Jp-GGG-Hp, intrigued us for a further investigation. We then respectively calculated the steric structure of these peptides by using a web protein analysis tool at http://bioserv.rpbs.univ-paris-diderot.fr/services/PEP-FOLD/, and viewed their 3D structures with the software of Pymol (http://www.pymol.org/). The results as shown in Fig. 1C indicated that both C20-Jp-Hp and C20-Hp-Jp exhibited possible intra-molecular interactions between the guanidinium group of argnine and the indole ring of tryptophan, where the *π-π* interaction as well as hydrogen bonding may occur, while these interactions were absent in other molecules. This difference might be associated with the comparatively higher antiviral activity of C20-Jp-Hp and C20-Hp-Jp than others, which will guide us for a further structural modification (Fig. 1C). Thus, it can be deduced that it is not necessary to have to be Jp domain for the antiviral activity of conjugated peptides. However, due to forming a favorable spatial structure contributed by the argnine residue from Jp domain, the antiviral activity of C20-Jp-Hp and C20-Hp-Jp might be enhanced in comparison with other congeners.

### Peptide C20-Jp-Hp reduces the replication of influenza virus HA protein

**Figure 2:**
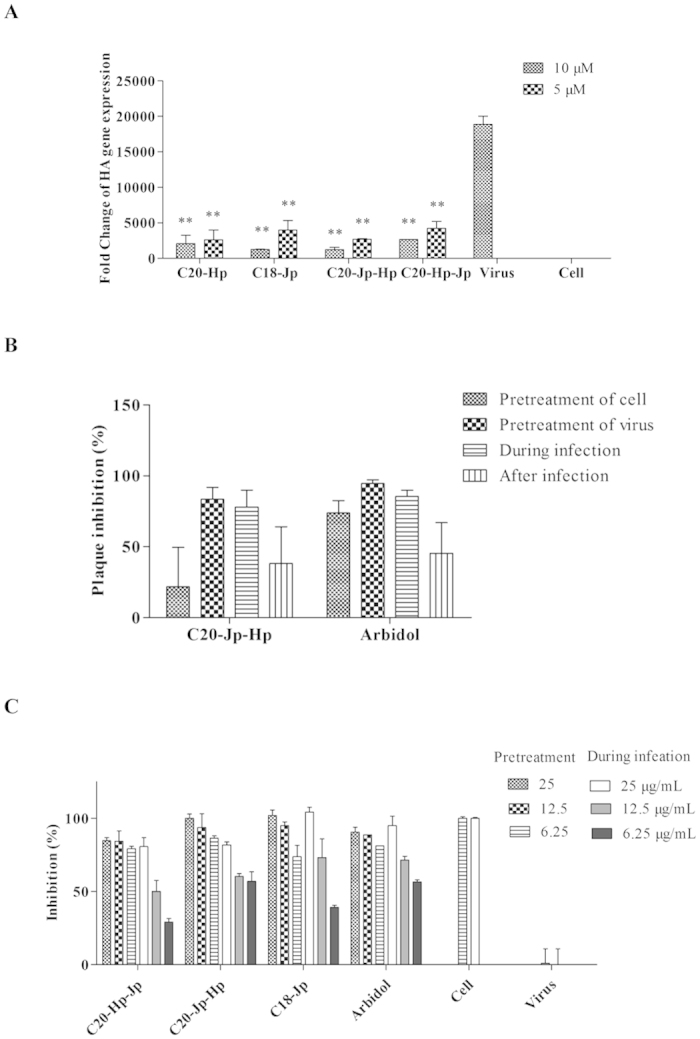
(**A)** The antiviral activity of selected peptides against influenza A/Puerto Rico/8/34(H1N1) by qRT-PCR. The peptides were pre-treated with A/Puerto Rico/8/34 virus at 100 TCID_50_ for 30 min, and then the virus-peptides mixture was transferred to the cells for another 1 h. At 24 h post-infection, the matrix gene was detected by quantitative real-time PCR. Statistical significance of the data with the virus group was defined as p  <  0.05 (*p  <  0.01, **p  <  0.001). (**B**) The plaque reduction assay of C20-Jp-Hp against influenza A/Puerto Rico/8/34(H1N1). The cells were divided into four groups. Group 1 (Pretreatment of cell): peptides were added to cell monolayers for 30 min before influenza virus adsorption at 37 °C under 5% CO_2_. Group 2 (Pretreatment of virus): peptides were pre-incubated with influenza virus for 30 min at 37 °C before added into the cells. Group 3 (During infection): virus A/PR/8/34 (100 TCID_50_) with peptide (10 μM) was simultaneously added to cells. Group 4 (After infection): peptides were added to cell monolayers after influenza virus adsorption at 37 °C under 5% CO_2_ for 30 min. After incubation at 37 °C 5% CO_2_ for 48 h, the plaques from each group were counted. Arbidol (5 μM) was used as positive control. (**C**) *In vitro* time-of-addition studies on anti-influenza viral activity of the C20-Jp-Hp and C20-Hp-Jp by CPE reduction assay in MDCK cells. Arbidol was used as positive control. Each data was performed in triplicate, and three independent determinations were carried out.

The initial results inspired us to further evaluate the inhibitory effects of these peptides on influenza virus replication. The influenza HA protein is associated with the virus replication, thus the mRNA levels of HA protein after treatment with peptides directly reflect the inhibitory effects of peptides^14^. As described in Fig. 2A, following the same procedure as the CPE assay, total RNA was extracted and reversely transcribed into cDNA. The real-time PCR was then performed with the SYBR Premix Ex Taq according to the manufacturer’s instruction. Influenza A/Puerto Rico/8/34 (H1N1) HA protein gene expression was normalized to cellular GAPDH gene. As a result, after treatment with tested peptide, the dramatically reduced mRNA expression level was observed (Fig. 2A), consistent with the results from the CPE assay.

### Hybridized peptides show lower cellular toxicity and higher therapeutic index than their prototypes

To assess the cellular toxicities of these peptides, MTT assay was then employed to quantitatively evaluate the viability of MDCK cells. As shown in Table 1, the CC_50_ values of C20-Hp-Jp and C20-Jp-Hp were 129.19 ± 3.85 and 135.54 ± 0.58 μM respectively, which were much higher than the prototypes of C18-Jp and C20-Hp, displaying promising potentials of these peptides in the development of anti-IAV agents. More importantly, the lower toxicity of hybridized peptides toward mammalian cells suggests a new strategy in the modification of lead peptides.

These data prompted us to further evaluate the toxicities by using other mammalian cells. As listed in Table 2, with the same procedure of MTT assay, the lower toxicity of hybridized peptides than their prototypes was further confirmed when tested with HaCaT cells, where *ca* two fold decreased toxicity of C20-Jp-Hp was observed in comparison with C20-Hp itself (Table 2).

### C20-Jp-Hp shows the inhibitory effect in the early stage of infection

To identify the detailed inhibitory step of peptide on influenza life cycle, the antiviral effect of C20-Jp-Hp toward A/Puerto Rico/8/34 (H1N1) viral strain was then studied with the plaque reduction assay by employing four different time points for drug administration: During infection, Pretreatment of virus, Pretreatment of cell and After infection^15^. Consequently, as shown in Fig. 2B, the Pretreatment of virus was represented as the most effective drug administration.

The early stage inhibitory effect of C20-Jp-Hp and C20-Hp-Jp was then confirmed by the CPE assay. The inhibition of virus *versus* various peptide concentrations of C20-Jp-Hp, C20-Hp-Jp and C18-Jp toward A/Puerto Rico/8/34 (H1N1) viral strain were performed by using two drug administrations (During infection and Pretreatment of virus). As a result, all peptides exhibited higher inhibitory effects with Pretreatment of virus than with During infection (Fig. 2C), consistent with the results from plaque reduction assay, thus indicating that these peptides inhibit the virus infection in the early stage by functioning as “entry inhibitors”, similar to their prototypes of C18-Jp and C20-Hp^8^,^9^.

### C20-Jp-Hp inhibits the entry of H5N1 influenza A pseudovirus

Due to the potent anti-IAV activity and the good selectivity index, we then employed H5N1 pseudovirus as a model to investigate the possible mechanism of action of this peptide. As reported previously^16^, the pseudo-typed virus was constructed by using the plasmids encoding HA and NA of A/Thailand/Kan353/2004 with HIV backbone, by which the antiviral effect was subsequently tested by measuring the inhibitory effect on the infection of H5N1 pseudovirus on MDCK cells.

**Figure 3:**
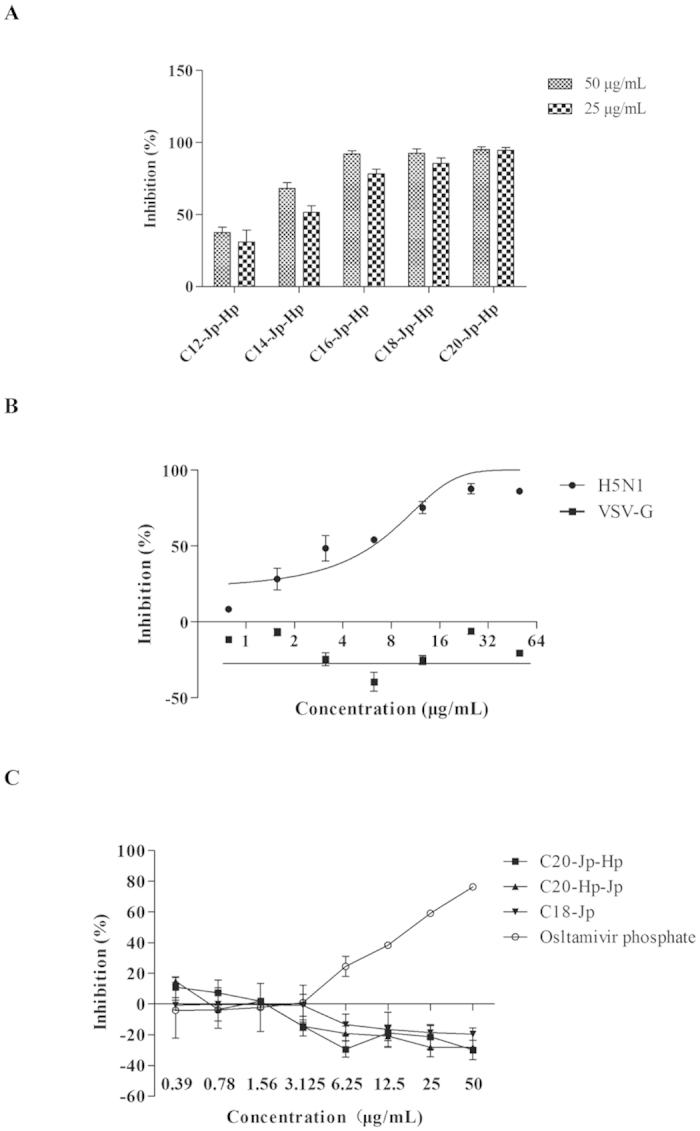
(**A**) Inhibition of the infectivity of H5N1 pesudovirus by Cn-Jp-Hp (n = 12, 14, 16, 18, 20) peptides. The peptides in various concentrations were incubated with pseudo-typed particles for 30 min at 37 °C, before transferred to the MDCK cells. The mixture of virus, peptide and MDCK cells was incubated for another 48 h, and then the luciferase activity corresponding to the survival of viruses was measured in a microplate luminometer. (**B**) The VSV-G pseudovirus was constructed by employing VSV-glycoprotein encoded plasmid similar to that of H5N1 pseudovirus. C20-Jp-Hp was serially two-fold diluted from 50 to 0.78 μg/mL in culture medium and then incubated with VSVG pseudovirus at 37 °C for 30 min prior to transferring into the MDCK cells. With the same procedure as that of H5N1 pseudovirus, the inhibitory effect toward VSVG pseudovirus was determined. As a comparison, C20-Jp-Hp at various concentrations inhibiting the infectivity of H5N1 pseudovirus was used in the same experiment. Each data was expressed as the mean of three independent replicates. (**C**) Neuraminidase (NA) inhibition assay. The neuraminidase from influenza A/Puerto Rico/8/34 (H1N1) virus was used in this experiment. The reaction mixture consisting of the tested peptides and virus in MES buffer was incubated for 45 min, and then 4-MU-NANA was added into each reaction well. The cleavage reaction was conducted for an additional 1 h, then terminated with 100 μL 34 mM NaOH (83% ethanol). The resulting fluorescence of the mixture was recorded at the excitation wavelength of 340 nm and emission wavelength of 440 nm. Oseltamivir phosphate was employed as positive control.

As shown in Fig. 3A, all Cn-Jp-Hp (n = 12, 14, 16, 18, 20) peptides with various lengths of lipid chain exhibited the inhibitory effect to a different extent, of which, C18-Jp-Hp and C20-Jp-Hp were proved to be the most active peptides against the tested viral strain. Obviously, the results indicate that these hybridized peptides may interact with glycoprotein of HA, NA or HIV backbone, thereby inhibiting the infectivity of viruses^17^.

### C20-Jp-Hp is unable to inhibit the entry of vesicular stomatitis (VSV) pseudovirus

Considering that both influenza A virus and vesicular stomatitis virus (VSV) take the same rout of endocytosis in the process of infection, we then employed VSV as a negative control to study the possible target of these peptides. The vesicular stomatitis pseudovirus was constructed by encoding VSV-glycoprotein plasmid into HIV backbone, similar to that of IAV pseudovirus, and then was applied to screen the selected peptides^18^.

As a consequence, C20-Jp-Hp didn’t show the ability to significantly reduce the infectivity of VSV pseudovirus on MDCK cells, indicating that the target of C20-Jp-Hp should not be the HIV backbone (Fig. 3B). Thus, the anti-IAV of these peptides may selectively interact with HA, NA, or both, to inhibit the infectivity of IAVs.

### C20-Jp-Hp is unable to inhibit the activity of neuraminidase (NA)

We then tested whether C20-Jp-Hp interacted with the neuraminidase (NA). An NA inhibition assay was thus employed to evaluate the enzymatic activity of NA, thereby to determine the possible target of this peptide^19^. By measuring the intensity of fluorescence resulted from the cleavage product of the substrate of 4-MU-NANA by the NA from influenza A/Puerto Rico/8/34 (H1N1) virus, the data in Fig. 3C showed that no inhibitory effect in the test range of 0.39 to 50 μg/mL was observed at all, indicating that C20-Jp-Hp was unable to inhibit the NA activity. Therefore, the most possible target of C20-Jp-Hp was that of HA by which to block the entry of virus into host cells.

### The anti-IAV activity of C20-Jp-Hp is associated with the interaction with HA2 subunit

**Figure 4:**
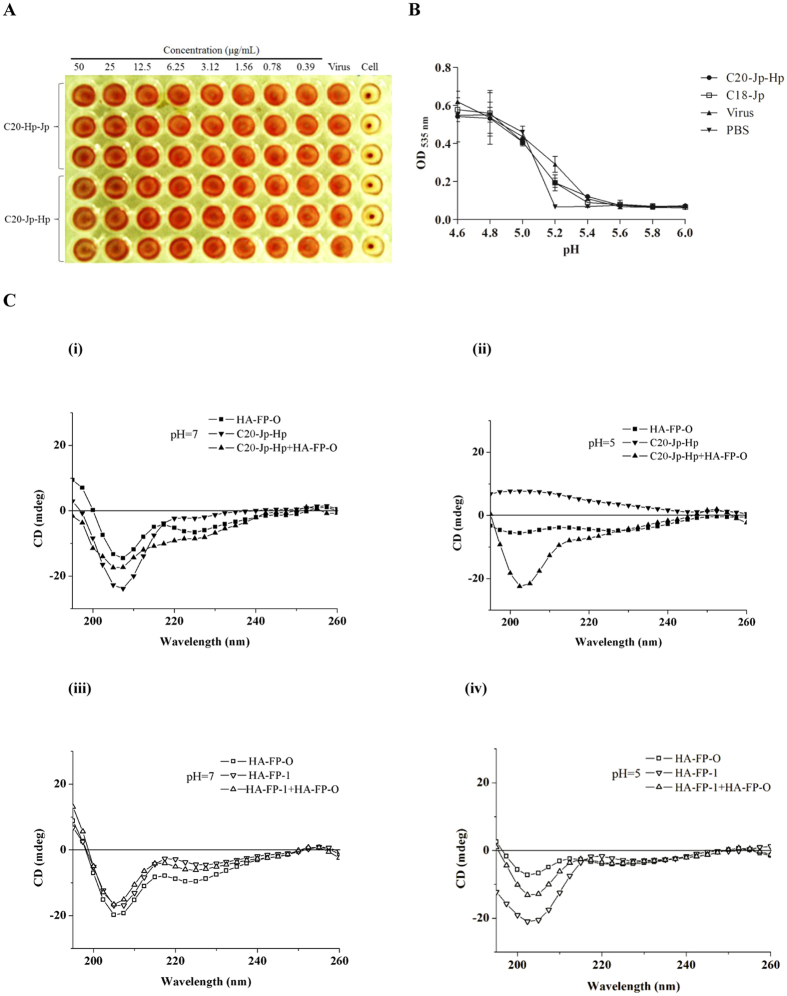
(**A**) The inhibitory effect of peptides on viral adsorption into target cells. Hemagglutination inhibition (HI) assay was performed using 4 times the HA units (4HAU) of virus per well. 25 μL of peptides from a twofold serial dilution in saline was added into 25 μL virus (4HAU), following the addition of 50 μL of erythrocytes (0.5% v/v in saline) into each well. The hemagglutination reaction results were read after incubation for 1 h. PBS without virus was used as positive control, while virus only as negative control. (**B**) Hemolysis inhibition assay. Peptide (10 μM) in PBS was mixed with the influenza virus A/PR/8/34 (H1N1) strain. 200 μL of 2% chicken erythrocytes pre-warmed at 37 °C was then added. After incubation at 37 °C for another 30 min, 100 μL of sodium acetate (0.5 M; pH 4.6–6.0) was added and mixed with the erythrocyte suspension to trigger hemolysis. After incubation for 30 min, the mixture was centrifuged and the supernatants containing released hemoglobin were measured at OD_535_. (**C**) The interactions between C20-Jp-Hp and HA-FP-O analyzed with circular dichroism (CD) spectra. (i) C20-Jp-Hp interacted with HA-FP-O in PBS (pH 7.4) and (ii) acidic condition (pH 5.0); (iii) HA-FP-O mixed with HA-FP-1 in PBS (pH 7.4) and (iv) in acidic condition (pH 5.0). CD curves of individual or mixed peptides with equimolar concentrations were scanned from 195 to 260 nm with an average of four scans.

As mentioned above, the HA glycoprotein is consisting of two subunits, HA1 and HA2. To characterize the possible interactions between C20-Jp-Hp and HA, we then carried out an HA inhibition (HI) assay to determine whether sialic acid binding site on HA1 subunit would be the possible target of C20-Jp-Hp^20^. As a result, no inhibition of agglutination of chicken erythrocytes was observed, indicating that sialic acid binding site on HA1 subunit was not the binding site of C20-Jp-Hp (Fig. 4A).

Next, we tested that whether HA2 subunit was the possible target of C20-Jp-Hp. In the events of virus-host cell membrane fusion, the HA2 undergoes conformational changes triggered by exposure to low pH in the endosome^21^. Thus, we employed a hemolysis inhibition assay to evaluate the inhibitory effect of C20-Jp-Hp on the lysis of erythrocyte induced by influenza virus of A/PR/8/34 (H1N1) under low pH. As shown in Fig. 4B, when 2% chicken erythrocytes was mixed with equal volume of peptide (20 μM) and influenza virus A/PR/8/34 (H1N1) in a 96-deepwell plate, the lysis of erythrocytes under acidic condition was decreased compared with the hemolytic effect of virus only. Thus, the antiviral activity of C20-Jp-Hp may result from the inhibition of the conformational rearrangements of HA2 subunit thereby interrupting the fusion of virus-host cell membranes.

To confirm this deduction, we then used a peptide of HA-FP-O derived from the N-terminal region of HA2 to study the interactions between HA-FP-O and C20-Jp-Hp^22^. The experiment was carried out by measuring the circular dichroism (CD) spectra of the HA-FP-O in the presence and absence of C20-Jp-Hp under the neutral and acidic conditions, respectively. The peptide HA-FP-O is the segment of HA2 of H1 subtype with the sequence of GLFGAIAGFI**E**NGW**E**GMI**D**G. It is a so called fusion peptide responsible for the membrane destabilization and fusion under acidic condition, thus playing an important role in virus entry into host cells^4^. In addition, we employed a positively charged derivative of HA-FP-O, named as HA-FP-1 with the sequence of GLFGAIAGFI**K**NGW**K**GMI**K**G, as a control to study whether this peptide has a similar effect^23^.

The CD spectroscopy indicated that the significant changes were observed upon addition of C20-Jp-Hp into HA-FP-O, especially at pH 5 (Fig. 4C), where the mixture of these two peptides was well formed a type II α-helical structure with minimum at 203 nm similar to the CD curves of poly(Pro)II^24^, which is commonly observed in globular proteins^25^. In contrast, this phenomenon was not observed between the interactions of HA-FP-O and its positively charged derivative of HA-FP-1. Therefore, the dramatic difference in CD spectra between the individual and mixed peptides confirmed that C20-Jp-Hp may interact with HA2, possibly the fusogenic region, supporting the notion that the HA2 subunit of the viral glycoprotein is the specific target of C20-Jp-Hp.

### The potential binding site of C20-Jp-Hp may be the fusogenic region of HA2

The CD spectroscopy and hemolysis inhibition assay suggested that C20-Jp-Hp may interact with the fusogenic region of HA2, by which to block the conformational change of HA2, and subsequently leading to the inhibition of virus entry. To demonstrate this process and identify a potential binding site for the C20-Jp-Hp, we then performed a computer-assisted modeling.

**Figure 5:**
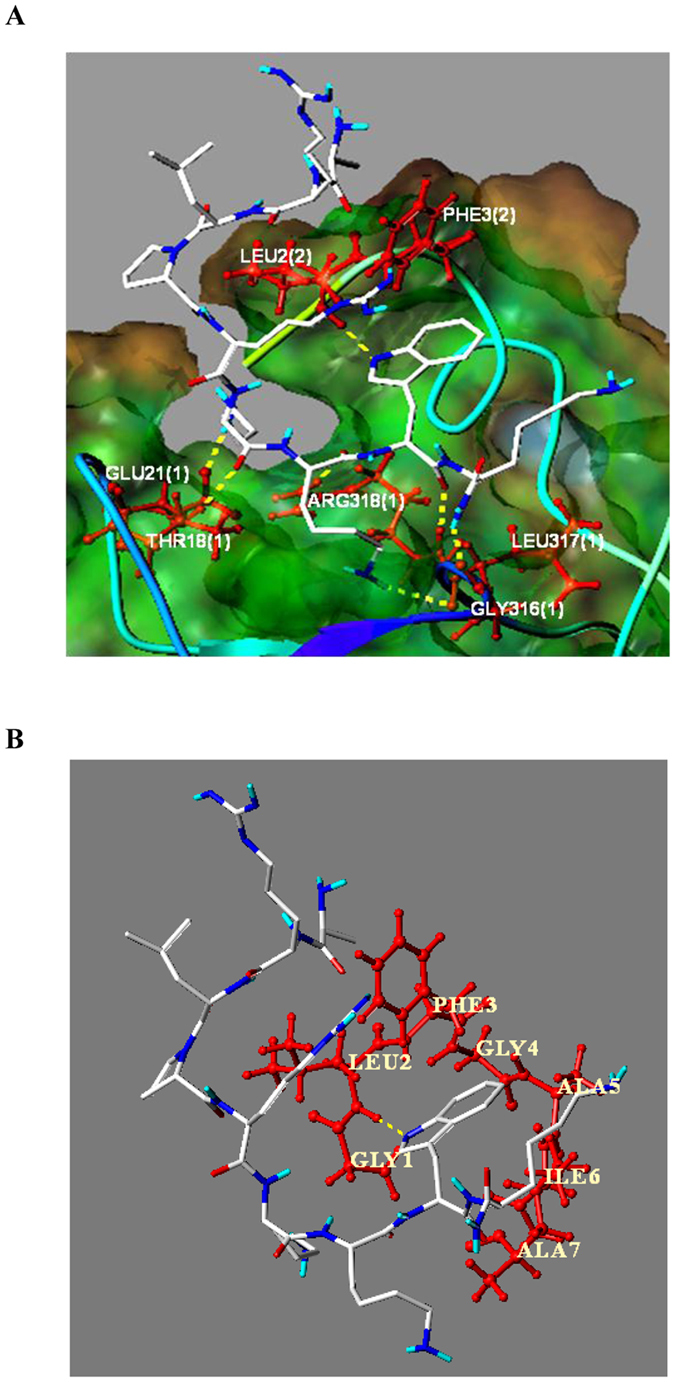
(**A**) The interactions between Jp-Hp and fusogenic region of HA2. (**B**) The partial structure of (**A**), where the interactions of Jp-Hp with the fusion peptide of HA2 was indicated. The docking simulation was performed in Sybyl 2.0 software, and the HA structure used was that of 4EDB (H1) adopted from the Protein Data Bank (PDB).

The docking simulation was carried out by using HA proteins of 4EDB (H1) and 4UO0 (H3) adopted from the Protein Data Bank (PDB) with the Sybyl 2.0 software. Considering the highly conserved structures of fusion peptides of influenza A viruses (Table 3)^26^, we thus performed the modeling on the fusogenic region of HA protein, a pocket embraced by the N- and C-terminal segments of HA1, and fusion peptide of HA2^27^,^28^. For comparison, both peptides Jp-Hp and Jp-GGG-Hp were employed for the docking simulation to compare their differences in the binding affinity. As a result, in the 4EDB protein, the total score of the interaction between Jp-Hp and the fusogenic region of HA2 was 5.9550, while for Jp-GGG-Hp, the score was only −0.3385. The same conclusion was also obtained in the protein of 4UO0, where the scores were 5.5110 (Jp-Hp) and 0.8572 (Jp-GGG-Hp), respectively. Further analyses showed that in the possible interaction complex formed by the fusogenic region of HA with the peptide of Jp-Hp (Fig. 5), the residues of Glu21(1), Thr18(1), Gly316(1), Leu317(1) and Arg318(1) form strong intramolecular hydrogen bonding with the residues from Jp-Hp (Fig. 5A). In addition, the hydrogen bonding between the nitrogen atom on the indole ring of tryptophan residue of Jp-Hp with NH group of Leu2(2), and the aromatic *π-π* stacking interaction between the indole ring of Jp-Hp and Phe3(2), the phenyl alanine residue of the fusion peptide of HA2, were also observed (Fig. 5B).

## Discussion

Due to the recent emergence of swine and avian flu, as well as of drug-resistant viral strains, new and effective anti-influenza drugs are urgently needed. In this paper, by hybridization of two short peptides, we identified a new group of potent anti-IAV lipopeptides with IC_50_ values in the range of 0.5 to 10.0 μM, of which, C20-Jp-Hp was represented as the most potent one when tested with a wide variety of influenza viruses in comparison with other analogues. Thus, C20-Jp-Hp was chosen as a lead compound for the following mechanism study.

Based on the pseudo-typed virus entry models, as well as the HA inhibition (HI) and hemolysis inhibition assays, the mechanism study indicated that C20-Jp-Hp may inhibit the viral infection in the early stage by interacting with the fusogenic region of HA2 subunit. This process involves the block of conformational rearrangements of HA2, thereby interfering with the membrane fusion of virus with targeting host cells. This deduction was further assessed by using CD spectroscopic technology, where a significant interaction between C20-Jp-Hp and the fusogenic peptide of HA-FP-O derived from HA2 was observed. This phenomenon was similar to the results reported by Li *et al*., who studied the interactions of a cholesterol-tagged peptide with the fusion glycoprotein of Newcastle disease virus (NDV), by which, a similar conformational change in CD spectrum was also observed^29^, supporting the conclusion proposed in this study.

Furthermore, from a structural point of view, the peptide of C20-Jp-Hp is a positively charged peptide with basic property, which may confer an additional protective effect by raising the intraendosomal pH, thereby preventing the conformational changes of HA2, similar to that of CL 61917 and CL 385319^30^.

Thus far, several influenza A virus entry inhibitors have been reported^31^,^32^,^33^. In this study, we present another one with the structure of C20-ARLPRKKWK. This compound is a lipopeptide acted by interfering with the fusogenic function, possibly interacting with the fusogenic region of HA2. The fusion peptides of influenza A viruses are known for their conservative sequences (Table 3) and a crucial role in the fusogenic process^26^,^27^,^28^,^29^,^30^,^31^,^32^,^33^,^34^, therefore, are a potential target for antiviral drugs to intervene. As a result, C20-Jp-Hp exhibits a broad and potent anti-IAV activity. In comparison with the prototypes of C18-Jp and C20-Hp, these hybridized peptides including C20-Jp-Hp exhibit a much lower cellular toxicities and higher selectivity indices, thus displaying a promising potential in the development of new anti-IAV drugs, which will lead us to a more vigorous and extensive *in vitro* and *in vivo* study in the future.

## Methods

### Material and chemicals

Chemicals of 9-fluorenylmethoxy carbonyl (Fmoc) protected L-amino acids, 2-(H-benzo triazole-1-yl)-1,1,3,3-tetramethyl uronium hexafluorophosphate (HBTU), Hydroxybenzotriazole (HOBt), and resin of rink amide 4-(2′,4′-dimethoxyphenyl-Fmoc- aminomethyl)-phenoxy acetamido-MHBA (MBHA) resin were purchased from GL Biochem Ltd. (Shanghai, China), while others including fatty acids, N,N-Diisopropyl ethylamine (DIEA) and organic solvents were purchased from Aladdin Co. (China) with peptide synthesis grade.

### Cells and viral strains

Madin Darby Canine Kidney (MDCK), HaCaT and 293T cells obtained from the American Type Culture Collection (ATCC) were grown in Dulbecco’s modified Eagle medium (DMEM) containing 10% fetal bovine serum (FBS). The influenza A/Aichi/2/68 (H3N2), A/Puerto Rico/8/34 (H1N1), A/FM/1/47 (H1N1) mouse adapted strain, A/Puerto Rico/8/34 (H1N1) with NA-H274Y mutation virus, clinical isolates of 690 (H3 subtype), 699 (H3 subtype), and the influenza B virus were propagated in the allantoic cavities of 9-day-old embryonated hen eggs at 37 °C. The allantoic fluid was harvested, clarified by low-speed centrifugation and stored at −80 °C. The virus titer was determined through the analysis of the 50% tissue culture infective dose (TCID_50_) on MDCK cells and evaluated by using Reed and Muench’s method^34^,^35^. The influenza A viruses 690 (H3), 699 (H3), and the influenza B virus were obtained from Guangdong Provincial Center for Disease Control and Prevention, which are clinically relevant viruses.

### Peptide synthesis

The methods for peptides synthesis in this study have been previously described^36^. Briefly, all peptides were synthesized by using standard 9-fluorenylmethoxy carbonyl (Fmoc) solid phase protocol on Rink Amide MHBA resin. The linear peptide sequences were assembled on an ABI 433A peptide synthesizer with 0.1 mmol scale. The synthesis was performed with eightfold excess of Fmoc-protected amino acid and catalyzed with eightfold excess of HBTU/HOBt and sixteen-fold excess of diisopropyl ethylamine (DIEA) in dimethylformamide (DMF). After peptide assembling, tenfold excess of fatty acid was added into the resin and subsequently coupled with standard amino acid coupling conditions. Then lipopeptides were cleaved from the resin using reagent M, which contains 87.5% trifluoroacetic acid, 2.5% ethanedithiol, 5% thioanisole and 5% deionized water. The cleavage reaction was conducted at room temperature for 3h, following standard work-up (crude product was precipitated in t-butyl methyl ether and washed twice with the same solvent). Peptide purity was then analyzed with RP-HPLC using a Waters HPLC with C18, 250 × 4.6 mm column (Sepax Technologies, USA) and purified if necessary. The HPLC analysis condition was as follows: flow rate, 1 mL/min; mobile phase, solvent A: water (0.075% trifluoroacetic acid), B: acetonitrile: methanol (v:v = 1:1, supplementary with 0.075% trifluoroacetic acid); gradient: 15% to 20% B (2 min), 20% to 60% B (10 min), 60% to 80% B (6 min), 80% to 90% B (6 min). All lipopeptides were found to have a purity of above 95%.

The molecular weight of each peptide was confirmed by electrospray ionization mass spectrometry (ESI-MS, Waters) as reported before^33^. Mass was performed using a Waters 3100 single-quadrupole mass spectrometer in the positive mode, specifically with a capillary voltage of −3 kV and cone voltage of −30 V. Nitrogen was used for both the cone gas (50 lh^−1^) and desolvation gas (650 lh^−1^), with the source and desolvation temperatures being held at 350 °C, respectively.

### Cytopathic effect (CPE) inhibition assay

The antiviral activities of peptides against influenza viruses were tested with CPE assay. MDCK cells were seeded into 96-well plates at 2 × 10^4^ cells per well and incubated at 37 °C in a humidified 5% CO_2_ atmosphere for 24 h prior to infection. A series of twofold diluted peptide concentrations were pre-incubated with influenza A virus (100 TCID_50_) at 37 °C for 30 min, and the virus-peptide mixtures were subsequently added to the cells and incubated for another 30 min. Then cells were washed twice with PBS to remove unabsorbed virus, followed by the addition of DMEM supplemented with 1 μg/mL TPCK-trypsin and 0.2% BSA. At 48 h post-infection, microscopy was performed to determine the antiviral effect, which was expressed as the concentration that reduced the virus-induced CPE by 50%. The data were further confirmed by using a MTT assay, and the resulting spectrophotometric data were used to calculate the IC_50_. The experiment was repeated at least three times and the antiviral drug of ribavirin was used as the positive control.

### Quantitative real-time PCR assay

The quantitative real-time PCR was employed to evaluate the inhibition of viral HA gene replication by peptides^9^. In brief, MDCK cells were grown with 2 × 10^5^ cells per well at 37 °C in 5% CO_2_ for 24 h until confluent. Then influenza A/Puerto Rico/8/34 (H1N1) virus at 100 TCID_50_ was pretreated with peptides at the concentration of 5 μM and 10 μM respectively at 37 °C for 30 min. Subsequently, with the same procedure as CPE assay, the total RNA was extracted with TRIzol reagent (Invitrogen) after 24 h post-infection, RNA quality and quantity were determined by UV spectrophotometer (Merinton SMA1000, US). The total RNA was reverse transcribed into cDNA using PrimeScript RT reagent kit. Real-time PCR was then performed in an ABI7500 PCR instrument (Applied Biosystems, US) with the SYBR Premix Ex Taq according to the manufacturer’s instruction. Influenza A HA protein gene expression was normalized to GAPDH gene, which is stably expressed in MDCK cells. Fold changes in gene expression were calculated using a classical 2^−ΔΔCT^ method.

The real-time PCR assay was conducted simultaneously to the cell treated with the peptides as described above. All samples were run in triplicate. qPCR conditions: 95 °C for 30 s, followed by 40 cycles of 95 °C for 5s, 60 °C for 34 s. The primer sequences for influenza virus HA gene were 5′-TTCCCAAGATCCATCCGGCAA- 3′ (forward) and 5′-CCTGCTCGAAGA CAGCCACAACG-3′ (reverse), GAPDH primers were used as internal control: 5′-AGGGCAATGCCAGCCCCAGCG-3′ (forward) and 5′-AGGCG TCGGAGGGCC CCCTC-3′ (reverse). Statistical significance of the data was determined by one-way ANOVA method using SPSS 20.0 software. Statistical significance of the data with the virus group was defined as p < 0.05 (*p < 0.01, **p < 0.001).

### Plaque reduction assay

2 × 10^5^ MDCK cells per well was seeded into 6-well plates and grown overnight until confluent, then the culture medium was removed^37^. Before the experiment, 100 TCID_50_ of A/PR/8/34 (H1N1) virus as well as peptide at 10 μM concentration in the culture medium was prepared. In this experiment, four types of drug administration were employed: group 1 (Pretreatment of cell): peptide was added to cell monolayers for 30 min before influenza virus adsorption at 37 °C under 5% CO_2_; group 2 (Pretreatment of virus): peptide in 10 μM was pre-incubated with influenza virus A/PR/8/34 (H1N1) for 30 min at 37 °C before added to the cells; group 3 (During infection): virus A/PR/8/34(H1N1) together with peptide (10 μM) was simultaneously added to cell monolayers; group 4 (After infection): peptide was added to cell monolayers after influenza virus adsorption at 37 °C under 5% CO_2_ for 30 min. Plates were incubated in a CO_2_ incubator for 1 hour with several intermittent rockings. After virus absorption, inoculums were removed and 3 ml of 0.5% agarose overlay prepared in culture medium containing 1 μg/mL trypsin and peptide was added into wells. After incubating for 48 h, the cell monolayer was fixed with 4% paraformaldehyde for 1 hour. Then agarose overlay was removed and cell monolayer was stained with 0.5% (w/v) crystal violet prepared in 5% ethanol. Plaques were counted by visual examination^37^.

### Cytotoxicity assay

The MTT assay was employed to evaluate the cytotoxicity of the peptides on MDCK and HaCaT cells. Mammalian cells (1 × 10^4^/well) grown in 96-well plate for 24 h were treated with peptides at the concentrations or blank control solutions (methanol of 3%) at 37 °C and 5% CO_2_ for 48 h. The viability of cells was then measured by the MTT assay. MTT (3-(4,5-dimethylthiazol-2-yl)-2,5-diphenyl tetrazo liumbromide) 0.5 mg/mL in DMEM was added into each well and incubated at 37 °C for another 4 h. Reduced MTT (formazan) was extracted with DMSO. Absorbance at the wavelength of 570 nm was read on a microtiter plate reader (Genios Pro Tecan, Swiss). Cytotoxicity of the peptides was estimated by comparison of the cell survival rate of peptides treated cells with that of solvent-treated. The survival rate of solvent-treated control was set as 100%.

### Measurement of the inhibitory activity on the entry of H5N1 pseudovirus

H5N1 pseudovirus was prepared by using the plasmids encoding HA and NA of A/Thailand/Kan353/2004 as previously described^9^. In brief, 1 μg HA plasmid, 1 μg NA plasmid, and 3 μg HIV backbone plasmid (pNL4-3.luc.R^−^E^−^), which contains an Env and Vpr defective, luciferase-expressing HIV-1 genome per well, were co-transfected into 293T cells in 6-well plate (60–70% confluent) using the PEI (polyethylenimine) as a transfection reagent. After incubation for 48 h, the culture supernatants were harvested and stored at −80 °C. The amount of pseudotyped particles was quantified using the luciferase assay.

In the process of measurement of the inhibitory activities of peptides, MDCK cells at 1 × 10^4^/well were grown 24 h until confluent. The peptides at indicated concentration were incubated with equal volume of pseudo-typed viral particles at 37 °C for 30 min before transferred to the cells and then incubated for another 48 h. To evaluate the antiviral effect, infected MDCK cells were subsequently lysed with the lysing reagent supplied by luciferase kit manufacture, followed by the addition of luciferase substrate. The luciferase activity was measured in a microplate luminometer (Genios Pro Tecan, Swiss).

### Measurement of the inhibitory activity on the entry of VSVG pseudovirus

By using a similar procedure as that of H5N1 pseudovirus, the VSV pseudovirus was constructed by employing VSV-glycoprotein encoded plasmid and HIV backbone plasmid (pNL4-3.luc.R^−^E^−^). The inhibitory activity of these peptides against VSVG pseudovirus was then tested as follows: MDCK cells (1 × 10^4^/well) were seeded in 96-well plates and grown overnight. The peptides were serially two-fold diluted from 50 to 0.78 μg/mL in culture medium and then incubated with equal volume of pseudo-typed particles at 37 °C for 30 min. Subsequently, the virus-peptides mixture was transferred into the MDCK cells and incubated for 48h at 37 °C before performing luciferase assay as described above.

### Neuraminidase (NA) inhibition assay

To investigate the mechanism of peptides, influenza virus NA activity was determined by measuring the intensity of fluorescence produced from the cleavage of the substrate of 4-MU-NANA [2-(4-methylumbelliferyl)-α-D-N-acetylneuraminic acid sodium] by the NA of the virus^19^. The neuraminidase from influenza A/Puerto Rico/8/34 (H1N1) virus was used for in this experiment. Briefly, the reaction mixture consisting of the tested peptides, virus in 33 mM MES buffer (containing 4 mM CaCl_2_, pH of 6.5) was incubated for 45 min at room temperature, and then 50 μL substrate of 4-MU-NANA dissolved in 33 mM MES buffer was added into each reaction well. The mixture was incubated for an additional 1 h at 37 °C covered with aluminum film, and then was terminated with 100 μL 34 mM NaOH (83% ethanol). Neuraminidase (NA) inhibitor of Osltamivir phosphate in 33 mM MES buffer was employed as positive control. The resulting fluorescence of the mixture was recorded at the excitation wavelength of 340 nm and emission wavelength of 440 nm^9^.


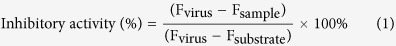


where F_virus_ is the fluorescence of the influenza virus control (virus, buffer and substrate), F_substrate_ is the fluorescence of the substrate control (buffer and substrate), and F_sample_ is the fluorescence of the tested samples (virus, sample solution and substrate). The experiment was repeated at least twice with a similar data each time.

### Hemagglutination inhibition (HI) assay

The HI assay was carried out to evaluate the inhibitory effect of peptide on viral adsorption into target cells. Four times of the HA units (HAU) of influenza A/Puerto Rico/8/34 (H1N1) virus per well (25 μL) in V-bottomed 96-well micro plates was prepared for the HI tests, then an equal volume (25 μL) of peptides started from 50 μg/mL of two-fold serial dilution in PBS was added into the plate. Subsequently, 50 μL of chicken erythrocytes (0.5% v/v in PBS) was added to each well to initiate the experiment. After incubation at 37 °C for 1 h, the hemagglutination reaction results were read as shown in Fig. 4B. PBS without virus was used as positive control, while virus only as negative control.

### Hemolysis inhibition assay

The inhibitory effect of peptide on the conformational changes of HA2 subunit was evaluated by hemolysis inhibition assay. The protocol was adopted from the previously reported literature with minor modification^22^. Briefly, 100 μL of peptide diluted in PBS was mixed with an equal volume of the influenza virus A/PR/8/34 (H1N1) strain (10^7^ TCID_50_/mL) in a 96-deepwell plate. After incubating the virus-peptide mixture at room temperature for 30 min, 200 μL of 2% chicken erythrocytes pre-warmed at 37 °C was added. The mixture was then incubated at 37 °C for another 30 min before inducing hemolysis.

To initiate the hemolysis, 100 μL of sodium acetate (0.5 M, pH 4.6 to 6.0) was added into the mixture to adjust the pH. After incubation for 30 min, cell debris and un-lysed cells were removed by centrifugation at 3000 rpm for 10 min and the absorbance of the released hemoglobin in the supernatant was read at 535 nm. The experiment was repeated at least twice with each data in triplicate each time.

### Circular dichroism (CD) spectra analyses

Peptides were dissolved in PBS (pH 7.4) and sodium acetate buffer (12.5 mM, pH 5.0) respectively. The scanning wavelength was used from 195 to 260 nm with an average of four scans in a spectropolarimeter of JASCO (J-810) by using cuvettes with 0.2 cm path length. On average, three independent scans were taken at a scanning rate of 100 nm/min. The CD data analyses were performed with the spectrum manager software provided by the equipment manufacturer.

To compare the changes in the secondary structure, the CD curves of each single peptide and the combination of two peptides (peptide mixtures) at an equimolar concentration in PBS (pH 7.4) or sodium acetate buffer (pH 5.0) were measured, respectively. In these studies, single peptides were prepared at a concentration of 0.2 mM, and peptide mixtures were prepared at equimolar concentrations (with respect to each peptide in mixture, the final concentration was 0.1 mM).

### Statistical analysis

The half cytotoxic concentration (CC_50_) and half inhibitory concentration (IC_50_) values of peptides were determined with Graph Pad Prism 5 (San Diego, CA), and the data were expressed as means ± standard deviation (SD) from triplicate assay with at least three independent experiments. Statistical significance of the data was determined by one-way ANOVA method using SPSS 20.0 software.

## Additional Information

**How to cite this article**: Lin, D. *et al*. A ‘‘building block’’ approach to the new influenza A virus entry inhibitors with reduced cellular toxicities. *Sci. Rep.* **6**, 22790; doi: 10.1038/srep22790 (2016).

## Figures and Tables

**Table 1:** The inhibitory effect of peptides on H1N1 influenza A virus and toxicity on MDCK cells.

Name	Sequence^a^	MW^b^	IC_50_ ± SD^c^ (μM)	CC_50_ ± SD^d^ (μM)	SI^e^
18-Jp	C18-ARLPR	878.03	1.44 ± 0.11*,g	114.33 ± 1.46	102.72
20-Hp	C20-KKWK	883.28	4.17 ± 0.04**	60.23 ± 0.74	14.41
Rirbavirin	NA^f^	244.21	12.85 ± 0.08**	NT	NA^f^
C16-Hp-Jp	C16-KKWKARLPR	1420.90	2.48 ± 0.24	>200	NA
C18-Hp-Jp	C18-KKWKARLPR	1448.76	0.75 ± 0.18	135.52 ± 1.29	178.49
C20-Hp-Jp	C20-KKWKARLPR	1477.01	0.71 ± 0.37	129.19 ± 3.85	180.01
C16-Jp-Hp	C16-ARLPRKKWK	1420.90	1.29 ± 0.40	>200	NA
C18-Jp-Hp	C18-ARLPRKKWK	1448.76	0.61 ± 0.04	139.59 ± 0.64	227.23
C20-Jp-Hp	C20-ARLPRKKWK	1477.01	0.53 ± 0.25	135.34 ± 0.58	253.03
C16-Hp-GGG-Jp	C16-KKWKGGGARLPR	1592.06	8.05 ± 0.52**	>200	NA
C18-Hp-GGG-Jp	C18-KKWKGGGARLPR	1619.92	6.99 ± 2.00**	>200	NA
C20-Hp-GGG-Jp	C20-KKWKGGGARLPR	1932.45	5.22 ± 0.37**	>200	NA
C16-Jp-GGG-Hp	C16-ARLPRGGGKKWK	1592.06	6.79 ± 0.17**	>200	NA
C18-Jp-GGG-Hp	C18-ARLPRGGGKKWK	1619.92	5.09 ± 0.35**	>200	NA
C20-Jp-GGG-Hp	C20-ARLPRGGGKKWK	1932.45	4.04 ± 0.02*	>200	NA
C20-ALLSA-Hp	C20-ALLSAKKWK	1338.84	1.69 ± 1.38*	>200	NA
C20-Hp-ALLSA	C20-KKWKALLSA	1338.84	3.80 ± 2.88*	>200	NA

^a^All C-termini were amidated;

^b^the molecular weight calculation was based on: http://www.peptidesynthetics.co.uk/tools/ ;

^c^The activity was tested with CPE assay toward influenza virus of A/Puerto Rico/8/34 (H1N1);

^d^The data was acquired with MTT assay against MDCK cells;

^e^SI: selectivity index;

^f^NT: not tested. NA: not available.

^g^^,*^Statistical significance was determined by one-way ANOVA method using SPSS 20.0 software. Values denote means with SD in five independent repeats. Statistical significance of the data with C20-Jp-Hp was defined as p < 0.05 (*p < 0.05, **p < 0.01, using ANOVA with Bonferroni test).

**Table 2:** Anti-influenza virus activities and cellular toxicities of selected peptides.

Name	IC_50_ ± SD (μM)^a^							CC_50_ ± SD (μM)^b^
	H3N2^c^	H1N1^d^	H1N1^e^	H1N1^f^	H3 (690)^g^	H3 (699)^g^	B^g^	HaCaT
C18-Jp	4.65 ± 1.24	11.55 ± 0.21	1.44 ± 0.11	3.24 ± 1.65	11.28 ± 4.86	4.29 ± 1.89	1.94 ± 1.51	40.59 ± 3.01
C20-Hp	4.62 ± 1.36	16.00 ± 3.72	4.17 ± 0.04	18.29 ± 4.10	5.43 ± 1.29	2.45 ± 0.37	4.53 ± 3.68	21.78 ± 0.25
Ribavirin	24.93 ± 1.10	17.11 ± 3.60	12.85 ± 0.08	NT^h^	8.47 ± 3.01	4.92 ± 1.45	6.71 ± 0.93	NT
C18-Hp-Jp	3.15 ± 0.93	5.25 ± 0.54	0.75 ± 0.18	NT	6.03 ± 2.96	NT	2.19 ± 0.29	37.45 ± 1.19
C20-Hp-Jp	2.97 ± 0.55	4.80 ± 0.66	0.71 ± 0.37	5.41 ± 1.39	2.47 ± 2.59	1.24 ± 0.08	1.31 ± 0.16*	33.72 ± 1.58
C18-Jp-Hp	1.63 ± 0.65	2.19 ± 0.43	0.61 ± 0.04	NT	3.55 ± 0.92	2.28 ± 0.50	3.60 ± 1.01	50.17 ± 2.94
C20-Jp-Hp	1.32 ± 0.31	1.71 ± 0.09	0.53 ± 0.25	1.59 ± 0.52	2.38 ± 1.66	1.95 ± 0.59	0.66 ± 0.60	41.48 ± 0.71
C20-ALLSA-Hp	10.51 ± 0.58	4.27 ± 3.42	1.69 ± 1.38	NT	NT	NT	NT	NT
C20 -Hp-ALLSA	8.90 ± 1.89	11.92 ± 6.19	3.80 ± 2.88	NT	NT	NT	NT	NT

^a^The data was obtained by CPE assay;

^b^The toxicity was evaluated with MTT assay;

^c^A/Aichi/2/68;

^d^A/FM/1/47 mouse adapted strain;

^e^A/Puerto Rico/8/34;

^f^A/Puerto Rico/8/34 with NA-H274Y mutation. Zanamivir was used as negative control, it IC_50_ toward A/Puerto Rico/8/34 with NA-H274Y mutation is 16.88 ± 2.43 μM and 0.31 ± 0.06 μM against A/Puerto Rico/8/34 (H1N1);

^g^690 (H3), 699 (H3), and influenza B virus were clinical isolates;

^h^NT: not tested.

**Table 3:** Alignment of fusion peptide sequences of influenza A virus strains^*^.

	1	2	3	4	5	6	7	8	9	10	11	12	13	14	15	16	17	18	19	20	21	22	23
H1	G	L	F	G	A	I	A	G	F	I	E	G	G	W	T	G	M	I	D	G	W	Y	G
H2	G	L	F	G	A	I	A	G	F	I	E	G	G	W	Q	G	M	V	D	G	W	Y	G
H3	G	L	F	G	A	I	A	G	F	I	E	N	G	W	E	G	M	I	D	G	W	Y	G
H4	G	L	F	G	A	I	A	G	F	I	E	N	G	W	Q	G	L	I	D	G	W	Y	G
H5	G	L	F	G	A	I	A	G	F	I	E	G	G	W	Q	G	M	V	D	G	W	Y	G
H6	G	L	F	G	A	I	A	G	F	I	E	G	G	W	T	G	M	I	D	G	W	Y	G
H7	G	L	F	G	A	I	A	G	F	I	E	N	G	W	E	G	L	V	D	G	W	Y	G
H8	G	L	F	G	A	I	A	G	F	I	E	G	G	W	S	G	M	I	D	G	W	Y	G
H9	G	L	F	G	A	I	A	G	F	I	E	G	G	W	P	G	L	V	A	G	W	Y	G
H10	G	L	F	G	A	I	A	G	F	I	E	N	G	W	E	G	M	V	D	G	W	Y	G
H11	G	L	F	G	A	I	A	G	F	I	E	G	G	W	P	G	L	I	N	G	W	Y	G
H12	G	L	F	G	A	I	A	G	F	I	E	G	G	W	P	G	L	V	A	G	W	Y	G
H13	G	L	F	G	A	I	A	G	F	I	E	G	G	W	P	G	L	I	N	G	W	Y	G
H14	G	L	F	G	A	I	A	G	F	I	E	N	G	W	Q	G	L	I	D	G	W	Y	G
H15	G	L	F	G	A	I	A	G	F	I	E	N	G	W	E	G	L	I	D	G	W	Y	G
H16	G	L	F	G	A	I	A	G	F	I	E	G	G	W	P	G	L	I	N	G	W	Y	G

*The H1 subtype is used as the reference sequence for this table, and changes from the H1 reference sequence are underlined^23^.
